# Seasonal changes and decrease of suicides and suicide attempts in France over the last 10 years

**DOI:** 10.1038/s41598-022-12215-3

**Published:** 2022-05-17

**Authors:** Marine Ambar Akkaoui, Christine Chan-Chee, Karine Laaidi, Gregory Fifre, Michel Lejoyeux, Guillaume Vaiva, Hugo Peyre, Pierre A. Geoffroy

**Affiliations:** 1 Département de Psychiatrie et d’addictologie, AP-HP, GHU Paris Nord, DMU Neurosciences, Hôpital Bichat - Claude Bernard, 75018 Paris, France; 2 Centre Psychiatrique d’Orientation et d’Accueil (CPOA), Hôpital Sainte Anne, GHU Paris Psychiatrie & Neurosciences, 1 Rue Cabanis, 75014 Paris, France; 3 Etablissement Publique de Santé Mentale de Ville Evrard, 202 Avenue Jean Jaurès, Neuilly Sur Marne, France; 4 Santé Publique France, 12 Rue du Val d’Osne, 94415 Saint-Maurice Cedex, France; 5 Météo-France, Direction des Services de la Météorologie, Avenue G. Coriolis, 31057 Toulouse, France; 6 GHU Paris - Psychiatry & Neurosciences, 1 Rue Cabanis, 75014 Paris, France; 7 Univ Lille, INSERM U 1772, CHU Lille, General Psychiatry Department, Hôpital Fontan, 59037 Lille, France; 8 Centre National de Ressources et Résilience pour les Psychotraumatismes Cn2r Lille Paris, Lille, France; 9 Neurodiderot, INSERM UMR 1141, Paris Diderot University, Paris, France; 10 Department of Child and Adolescent Psychiatry, Robert Debré Hospital, APHP, Paris, France; 11 Université de Paris, NeuroDiderot, Inserm, 75019 Paris, France; 12 CNRS UPR 3212, Institute for Cellular and Integrative Neurosciences, 67000 Strasbourg, France

**Keywords:** Epidemiology, Psychiatric disorders

## Abstract

The objective of this study was to examine the evolution of suicides and suicide attempts over the last 10 years in France. We analyzed the number of deaths by suicide and suicide attempts in metropolitan France (2009–2018) from French national databases, on a daily, weekly or monthly basis. Seasonal variation in suicide rates was modeled using a cosinor function. Based on this model, we determined the association of suicides and suicide attempts with geographic characteristics, age, gender, means used and psychiatric disorders. The number of suicides and suicide attempts decreased over the last ten years in France (mean decrease of 14.49% and 11.69% per year). We observed a significant and recurrent seasonal pattern of suicides and suicide attempts in France, with a peak in spring. The suicide and suicide attempt rates were higher in the northern departments of France. Suicides were more frequent for men (75%) and middle-age individuals (45–54 years old), while suicide attempts were more frequent for women (62%) and young adults and middle-age individual (15–19 and 40–49 years old). Nearly two-thirds of the patients who attempted suicide suffered from comorbid psychiatric disorders. Mood disorders was the most frequent comorbid psychiatric disorder (54%). Voluntary drug intoxication was the most common means of suicide attempt (80%), and hanging was the most common means of suicide (54%). The number of suicides and suicide attempts decreased in France over the last 10 years, with large and recurrent seasonal variations. These findings could be used to alert and adjust prevention policies, as well as developing preventive strategies such as chronotherapeutics.

## Introduction

Every year, more than 800,000 people die by suicide worldwide, which would correspond to about 1.5% of the total number of deaths^1^. In France, this proportion seems to be broadly similar, since it has been found that about 10,000 people die by suicide each year, representing up to 1% of all deaths^2^. Suicide is the leading cause of death among 15–29 years old and accounts for up to 50% of violent deaths worldwide^3^. It is estimated that there are about ten to twenty times more suicide attempts than suicides each year in the world^4^. The main risk factor for suicide is the existence of a history of suicide attempt. Indeed, among people who attempt suicide, it has been estimated that 1.6% die by suicide within 12 months and 3.9% within 5 years^5^. Suicide and suicidal behaviors are multifactorial phenomenon, involving biological and environmental factors, and are difficult to predict and to prevent^4^. For instance, three out of four people who die of suicide have contact with primary care physicians in the year preceding suicide, and about half of them within a month^6^. In this context, there is a clear need for better identification of robust prodromal signs of suicide and suicidal attempt, as well as effective preventive public health interventions.

In France, the National Public Health Agency (Santé Publique France) has implemented an epidemiological surveillance for suicide attempts and suicides in France. The objective of this study was to examine the evolution of suicides and suicide attempts over the last 10 years in metropolitan France.

## Methods

### Study design and population

We examined the number of death by suicide and suicide attempts in metropolitan France for the last 10 years (2009–2018) in French national databases provided by Santé Publique France. As the national public health agency, Santé Publique France has full legal access to national health databases^7^.

More specifically, the data on deaths by suicide and suicide attempts were provided on a daily basis, from two databases:

- A database for suicide attempts, with data from January 1, 2009, to December 31, 2018, grouped by department (French local administrative division). These data corresponded to all medical visits (consultations, hospitalizations, emergencies) for suicide attempts over the period of the study. These data were collected from the database using PMSI-MCO (*Programme de Médicalisation du Système d’Information—Médecine Chirurgie Obstétrique*) coding and correspond to ICD-10 codes X60 to X84 (detailed below).
- Deaths by suicide were extracted from the death certificates database (CepiDc-Inserm, medical research institute in France). These were daily data, by departments, over a period from January 1, 2009, to December 31, 2015. Data after this date were not available at the time of the extraction for analysis.

For both databases (suicide attempts and deaths by suicide), Santé publique France also provided information concerning age (5-year age groups), gender, and the method used for suicide in 10 categories: drug self-intoxication (X60, X61, X62, X63, X64), intoxication by other products (alcohol, solvents, gas, pesticides, chemicals) (X65, X66, X67, X68, X69), self-inflicted injury by exposure to smoke, flames and gas (X75, X76, X77), self-inflicted injury by sharp object (X78, X79), self-inflicted injury by hanging, strangulation, suffocation (X70), self-inflicted injury by jumping (X80), self-inflicted injury by firearm (X72, X73, X74), self-inflicted injury by drowning or submersion (X71), self-inflicted injury by intentional collision (X81, X82), self-inflicted injury by unspecified means (X83, X84).

These data were merged with a third database from PMSI-MCO 2009–2018 providing the psychiatric disorders that had been identified at the time suicide attempts. These psychiatric diagnoses were coded according to ICD-10 criteria, and correspond to the following diagnoses : organic, including symptomatic, mental disorders (F00, F01, F02, F03, F04, F05, F06, F07, F09), mental and behavioral disorders due to psychoactive substance use (F10, F11, F12, F13, F14 ,F15, F16, F17, F18, F19), psychotic disorders (F20 (schizophrenia), F21, F22, F23, F24, F25, F28, F29), bipolar disorders (F30, F31), depressive disorders (F32, F33, F34, F38, F39), neurotic, stress-related and somatoform disorders (F40, F41, F42, F43, F44, F45, F48), eating disorders (F50) and disorders of adult personality and behavior (F60, F61, F62, F63, F64, F65, F66, F68, F69). In France, the CepiDC death certificate database is not sufficiently informative about comorbid psychiatric disorders at the moment of the death, and psychological autopsies are rare and only performed on request. Therefore, the associations with psychiatric comorbidities will only be studied in this work with suicide attempts, which are coded by psychiatrists.

We calculated a rate of suicide and suicide attempts per department and year, using the number of inhabitants per department and year provided on a database available on the INSEE website (https://www.insee.fr; National institute for statistics and economic studies).

All the methods were performed in accordance with relevant guidelines and regulations.

### Analysis

Daily data were grouped by week or month. Suicide rates were calculated by department per 100,000 inhabitants, per day, week and month. We made a statistical description of the different variables. We statistically examined the seasonal effect of deaths by suicide and suicide attempts by using the cosinor model, which captures a seasonal pattern using a sinusoidal wave (analysis made with the “Season” R package, made by Barnett & Dobson).

Analyses were carried out using R Studio software, version 1.1.463.

## Results

The complete observation period was of 3652 days for suicide attempts and of 2556 days for deaths by suicide, reporting 68,316 deaths by suicide and 913,938 suicide attempts.

### Demographic data

**Figure 1:**
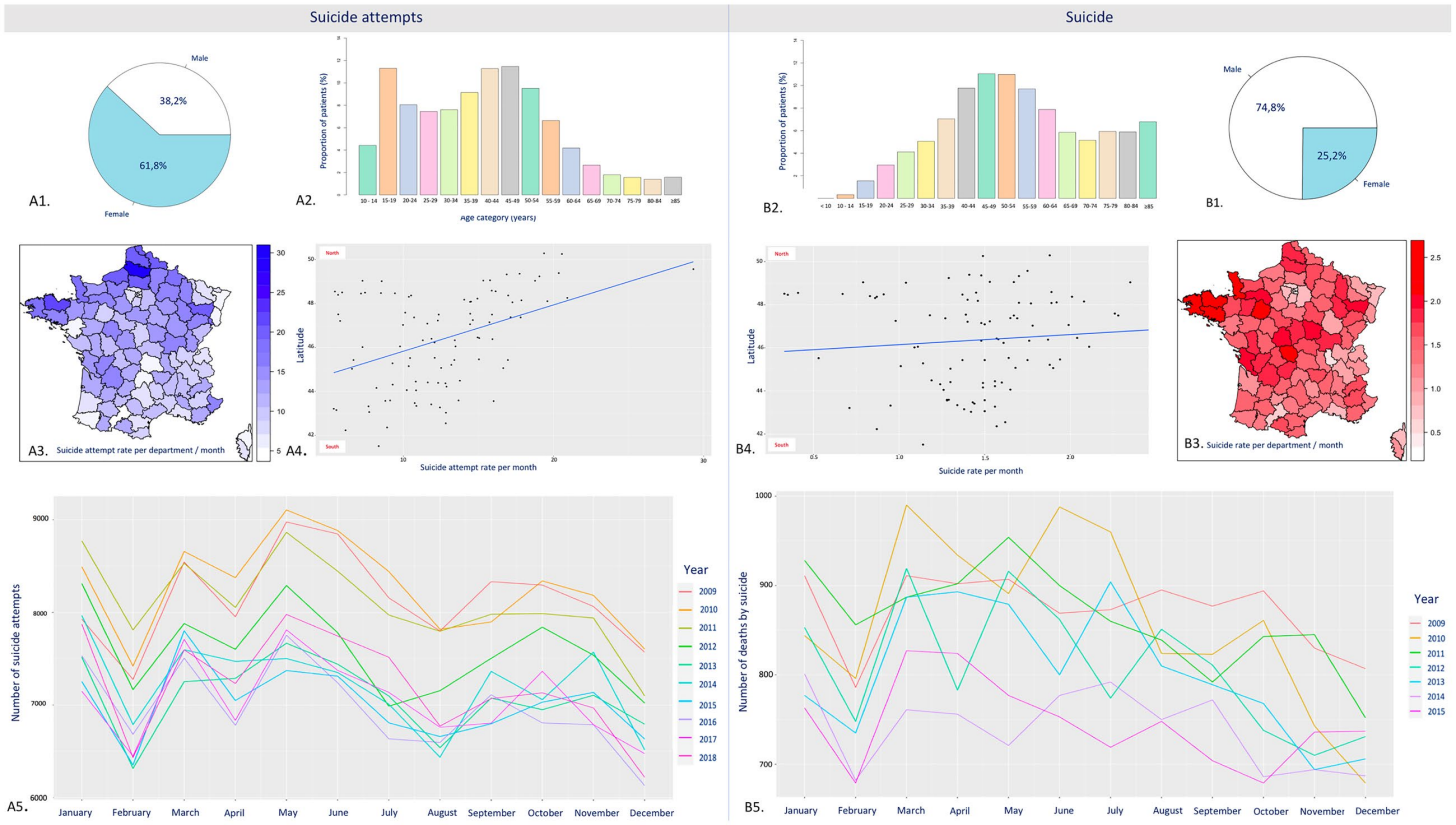
Pie chart representation of gender distribution in the study population. (**A1**) Suicide attempts over the 2009–2018 period; (**B1**) Suicides over the 2009–2015 period. Bar graph representation of the age distribution (5-year age groups) of patients admitted for suicide attempt (**A2**) or suicide (**B2**). Representation of the suicide attempt rate (**A3**), and the suicide rate (**B3**) per month and per 100,000 population, over the period 2009–2018 for suicide attempts and over the period 2009–2015 for suicides. Maps were created using R Studio software, version 1.1.463, with the library “raster”. Suicide attempt rate (**A4**) and suicide rate (**B4**) by month, based on department latitude barycenter. Number of suicide attempts (**A5**) and death by suicide (**B5**) per month in metropolitan France.

Patients who attempted suicide were mostly female [564,338 women (74.8%) and 349,600 men (25.2%)]. An opposite ratio was observed for deaths by suicide, with mostly men (74.8%, n = 51,148) compared to women (25.2%, n = 17,168) (Fig. 1A1,B1).

Suicide attempts were more frequent in younger patients, with two peaks: 15–19 years old and 40–49 years old. Deaths by suicide increased progressively with age, with a peak between 45 and 54 year-old and with a flattening at older ages see Fig. 1A2,B2).

### Methods of suicide

**Figure 2:**
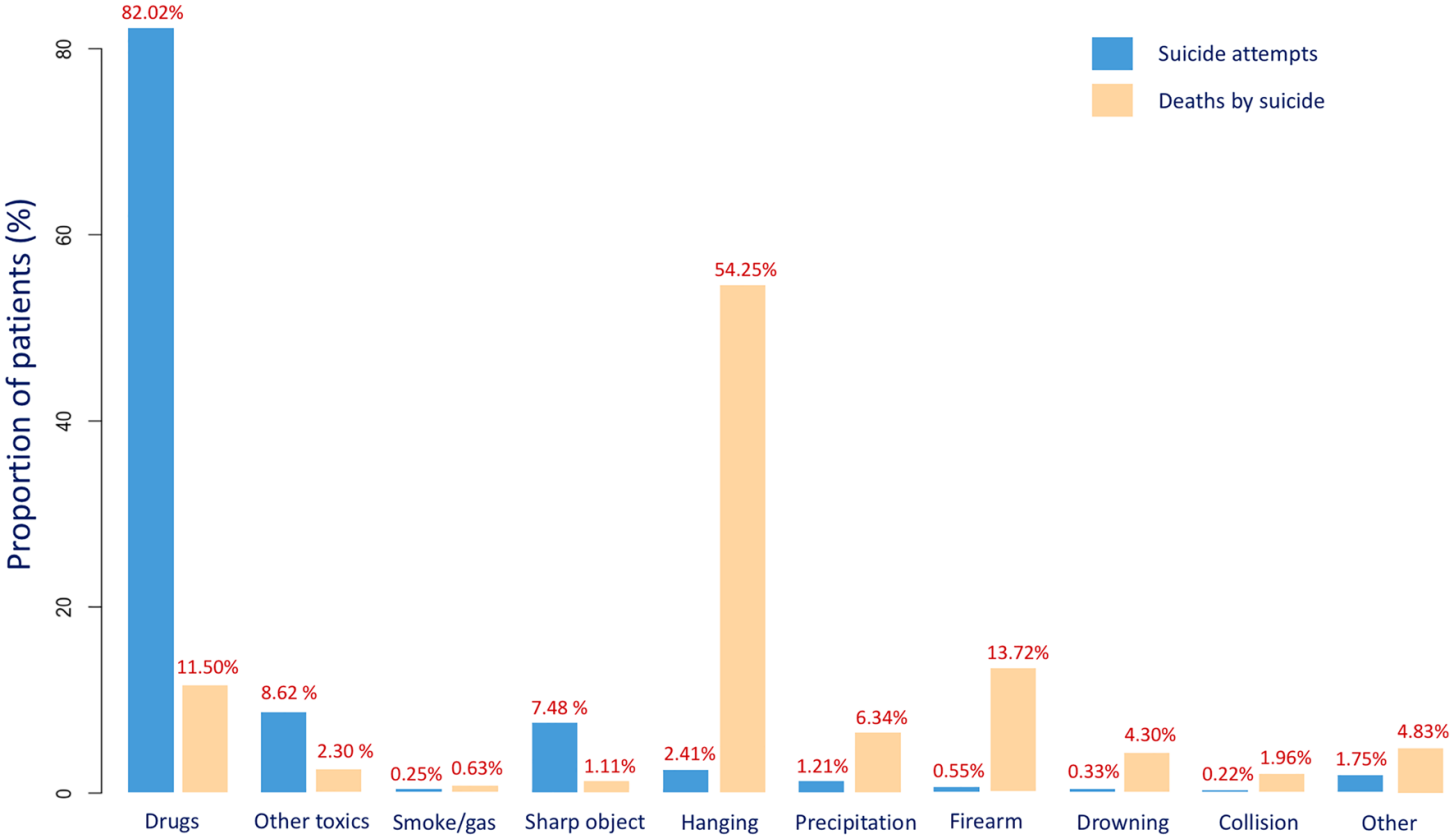
Bar chart representation of the suicidal means used in France, over the 2009–2018 period for suicide attempts and over the 2009–2015 period for suicides.

The most frequent mean used for suicide attempts was self-administered medication (82.02%, n = 749,655). Other most frequent means were self-intoxication by other toxics (8.62%, n = 78,804) and self-inflicted sharps injuries (7.48%, n = 68,407). For deaths by suicide, the most common mean was self-inflicted injuries by hanging (54.25%, n = 37,066), by firearms (13.72%, n = 9375), and self-intoxication by drugs (11.5%, n = 7979) (Fig. 2).

### Suicide and suicide attempt rates

The average number of suicides and/or suicide attempts per day per department in France was 3.068 over the entire study period.

The department with the highest rate of suicide attempts per month per 100,000 inhabitants was the Somme in the North (6.63 suicide attempts per week per 100,000 inhabitants). Other departments with the highest rate of suicide attempts per week per 100,000 inhabitants were: Côte d'Armor, Manche, Finistère, Seine Maritime (North-West), Nord, Pas de Calais, Oise (North), Vosges and Territoire-de-Belfort (North-East).

The department with the highest suicide rate per 100,000 inhabitants per month was the Côte d'Armor in the North-West (0.57 suicides per 100,000 inhabitants/week). Other departments with the highest weekly suicide rate per 100,000 inhabitants are Manche, Morbihan, Finistère, Sarthe, Orne (North-West), Charente Maritime (West), Creuse, Indre (Center), Nièvre (East) (Fig. 1A3,B3).

Detailed rates of suicide and suicide attempts per day, per week, and per month for each department in metropolitan France are given in Table S1 in Supplementary Material.

Suicide rates and suicide attempts rates are higher in the northern departments of France than in the south. Figure 1A4 and B4 shows the rates of suicide attempts (A4) and suicide (B4) by month, based on the latitude barycenter of the department. A regression curve was plotted, showing an increase in suicide and suicide attempts rates with latitude. This tendency is much more evident for suicide attempts than for suicides.

### Psychiatric disorders

On average, 63.27% of suicide attempts were associated with a diagnosed psychiatric disorder. Mood disorders were the most frequent psychiatric disorders associated with suicides and suicide attempts. Then, substance use disorders and personality disorders were also often associated with suicide attempts (Table 1).

**Table 1:** Average number of psychiatric disorders associated with suicides and suicide attempts per year.

ICD-10 code	Corresponding disorders	Suicide attempts (2009–2018)		
		Female	Male	Total
F0	Organic, including symptomatic, mental disorders	565.6	440.4	1006
F1	Mental and behavioral disorders due to use of alcohol	10,314.5	11,630.5	21,945
	Mental and behavioral disorders due to other substances	2878	3671.5	6549.5
F2	Schizophrenia	391	785	1176
	Other psychotic disorders	734.5	775.6	1510.1
F3	Bipolar disorders	1603.1	712.4	2315.5
	Depressive disorders	19,334.3	10,134.6	29,405.9
F4	Neurotic, stress-related and somatoform disorders	6703.1	3219.2	9922.3
F5	Eating disorders	610.2	41.1	652.3
F6	Disorders of adult personality and behavior	2209.8	1300.7	3510.5
	Total	35,063.4	27,483.2	58,761.1

*ICD-10* International Classification of Diseases, 10th version.

### Evolution of the number of suicides and suicide attempts over the years

**Figure 3:**
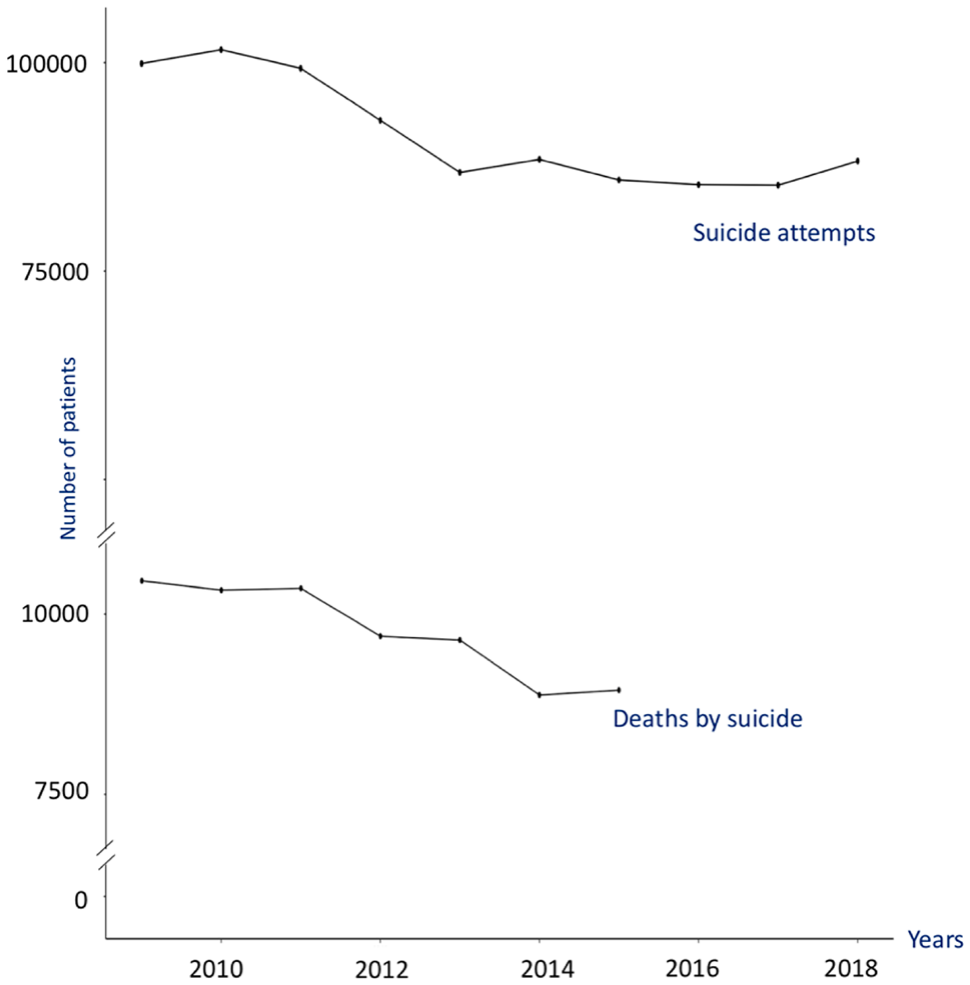
Evolution of death by suicide and suicide attempts over the years 2009–2018.

Figure 3 shows the evolution of the number of suicides and suicide attempts over the years.

The number of suicides and suicide attempts has decreased over the last ten years. Before 2012, the number of suicide attempts was over 100,000 and the number of suicide was over 10,000. After 2012, a continuous decrease was observed with 90,000 suicide attempts (2018) and less than 9000 suicides (2015). Thus, between 2009 and 2015, the total number of suicides decreased by 14.49%, and the total number of attempted suicides decreased by 11.69%.

### Seasonality of suicide attempts and suicides

Figure 1A5 and B5 shows the evolution of the number of suicide attempts and the number of suicides according to the month of the year, and shows a seasonal pattern of suicidal behaviour that replicates itself every year over the 10 years. A decrease is observed in the number of suicide attempts in the winter (off-peaks in December and-February), with an increase in early spring (April–May). The peak of suicide attempts is reached in May–June, before decreasing to a minimum in August. There is also a drop in the number of visits to the emergency room for suicide attempts during the last month of the year (mainly the last week).

Using an adjusted significance level of 0.025, we found a significant seasonality effect with the cosinor function, for the numbers of deaths by suicide and suicide attempts for each year during the 10 years follow-up (p < 0.0001). The period of months with the highest number of events was 4.3 (i.e. between April and May) for suicide attempt, and 5.1 (i.e. at the beginning of May) for suicide, and the period with the lowest number of events was 10.3 (i.e. between October and November) for suicide attempt and 11.1 (i.e. at the beginning of November) for suicide.

## Discussion

This study reports two important observations. First, a decrease of suicides and suicide attempts occurred in France since 2010. Secondly, a seasonality is repeatedly observed over years, regarding both suicides and suicide attempts, with a peak in spring.

Regarding the decrease of suicides and suicide attempts from 2010, it may be the consequence of several suicide prevention programs launched in France and worldwide since 2000. The four axes of suicide prevention defined by the Ministry of Social Affairs and Health^8^ are: promoting prevention, reducing access to lethal means, promoting access to healthcare, and improving knowledge of the epidemiological situation^9^. In a review of the literature of efficient interventions on suicide prevention, the three most efficient categories of interventions were the limitation of access to lethal means, the preservation of contact with the patients hospitalized for a suicide attempt after hospitalization, and the implementation of emergency call centers^10^. Over the last 10 years in France, this kind of measures to prevent suicidal repetitions have been implemented, through postcards, recalls and home visits^11^. These interventions have shown a tendency to reduce suicidal repetitions^11^. In addition, the VigilanS system has been implemented in many regions in France, combining brief contact interventions and a sanitary monitoring of suicide attempts^12,13^.

The repeated seasonal peak in suicidal behavior observed in spring and early summer in France confirmed in France previous observations from other international studies^14–17^. A possible pathophysiological hypothesis is that after cold nights during spring and early summer, the warming of the human body during the day would cause hyper activation of the brown fat tissue, causing increased anxiety and mental activity, and promoting suicidal behavior^18–20^. Another possible mechanism would be that the increase in temperature could promote violent behavior and impulsivity by increasing serotonin levels^21–25^. An Australian study reported decreased serotonin levels in winter, and increased levels in summer, suggesting a sensitivity of serotonin to temperature and duration of sunshine^21^. These hypotheses are also consistent with our observations of a lowest rate of suicides and suicide attempts in February, corresponding to the time when the temperature is the coldest. However, we observed a second lowest rate in the number of suicides and suicide attempts in August. This drop in the number of suicidal behaviors can be explained in part by the fact that the month of August corresponds to the major school vacations for the majority of French people, which means time spent with family or friends, which is a protective factor against suicidal behavior^4^. Indeed, the increase of temperature, including at very elevated temperatures, rather leaded to a plateau of increased suicidal behaviors^26,27^. The same hypothesis could be made for the fall of the month of December, corresponding to the end of year celebrations and family reunions. Finally, the photoperiod changes in spring, with an increase of daylight length, may also lead to suicidal behaviors as a consequence of circadian rhythm and sleep–wake cycle alterations^28^. Further studies are expected to better understand underpinning physiological mechanisms of the meteorological factors impact on suicidal behaviors.

These findings highlighting a clear seasonal pattern of suicidal behaviors warrant to impact the existing public health measures mentioned previously, such as reinforcing the link during the spring and summer with patients with a history of suicide attempts or suicidal thoughts, via brief contact interventions for instance^13,29^ In addition, broad public health campaigns of prevention and awareness should be delivered during the at-risk period. At the individual level, practitioners should be more vigilant during the spring and summer for at-risk patients with suicidal ideation, or history of suicidal behaviors, or a psychiatric disorder at high risk of suicide.

Approximately one-third of patients who committed suicide and two-thirds of patients who attempted suicide had an associated psychiatric disorder. The most common psychiatric disorder was mood disorders. Those with a substance use disorder were more likely to commit suicide attempts, but not completed suicides. Psychiatric disorders are one of the most important risk factors for suicide^4,29,30^. As such, about one-third of patients with bipolar disorder will attempt suicide in their lifetime^31^. Moreover about one quarter of patients with bipolar disorder have a seasonal pattern of their depressive recurrences, and one out of six patients present with seasonal manic recurrences^32,33^. Patients with schizophrenia are up to 20 times more likely to die by suicide than the general population use^34^. A particular shared vulnerability between suicidal behaviors and psychiatric disorders has been confirmed in our study. This observation calls for systematically screen for suicidal behaviors in these at-risk psychiatric populations, may be at particular at-risk periods with targeted public health interventions or programs.

### Limitations

Some limitations should be acknowledged. First, the suicide database extracted from the death certificates has an underestimation of about 10% for the national data, due in particular to deaths for which the cause remains undetermined or to those for which a forensic examination has been carried out and the conclusion has not been reported^35^. Similarly, a probably non-negligible and difficult to estimate number of suicide attempts are not medicalized, and therefore have not been recorded in our databases. However, this rate of non-medicalized suicide attempts seems to be stable over time^36^. This is also a common caveats, already reported by numerous studies examining suicide behaviors. Second, only two-third of patients who attempted suicide had a reported psychiatric comorbidity. This rate suggests that either a significant proportion of patients with suicidal behavior have no psychiatric history or that such history is under-diagnosed or sought in practice. It may also be that there is a lack of coding or reporting of these psychiatric disorders in hospital departments. In addition, we were unable to explore psychiatric comorbidities associated with deaths by suicide because of a lack of information on the death certificates. A review about psychological autopsy studies estimated that more than 90% of suicide deaths are associated with a comorbid psychiatric disorder^37^. This under-reporting / under-coding of psychiatric comorbidities after a suicide attempt or a death by suicide shows that there is a need to improve the screening for associated psychiatric disorders in France. In our analyses, we were interested in the distribution of the different psychiatric disorders rather than their prevalence. Despite these limitations, this work presents a good epidemiological synthesis of the state of suicide and suicide attempts in France over the last years.

## Conclusion

The number of suicides and suicide attempts seems to have decreased in France over the last 10 years, suggesting the effectiveness of the public health prevention measures. A seasonality in suicidal behaviors exist with a peak in spring, which requires further studies to better understand underpinning physiological mechanisms and the different meteorological factors that may influence these variations.

## Supplementary Information

Supplementary Table S1.

## Data Availability

Data available on request from the authors.
