# Supplementary material for: Seasonal changes and decrease of suicides and suicide attempts in France over the last 10 years
Source: Sci Rep. 2022 May 17;12:8231. doi: 10.1038/s41598-022-12215-3 (PMC9114420; doi:10.1038/s41598-022-12215-3)
Supplement: Supplementary file 1 — Supplementary Table S1. [file 41598_2022_12215_MOESM1_ESM.docx]

| **Number** | **Department** | **Suicide** | | | **Suicide attempts** | | |
| --- | --- | --- | --- | --- | --- | --- | --- |
|  |  | **Days** | **Weeks** | **Months** | **Days** | **Weeks** | **Months** |
| **01** | **Ain** | 0,04 | 0,25 | 1,11 | 0,32 | 2,21 | 9,77 |
| **02** | **Aisne** | 0,06 | 0,38 | 1,70 | 0,55 | 3,82 | 16,88 |
| **03** | **Allier** | 0,05 | 0,36 | 1,61 | 0,40 | 2,73 | 12,07 |
| **04** | **Alpes-de-Haute-Provence** | 0,05 | 0,37 | 1,65 | 0,34 | 2,31 | 10,22 |
| **05** | **Hautes-Alpes** | 0,05 | 0,36 | 1,60 | 0,40 | 2,78 | 12,31 |
| **06** | **Alpes-Maritimes** | 0,04 | 0,29 | 1,28 | 0,53 | 3,62 | 16,01 |
| **07** | **Ardèche** | 0,05 | 0,35 | 1,56 | 0,36 | 2,45 | 10,82 |
| **08** | **Ardennes** | 0,05 | 0,33 | 1,48 | 0,58 | 4,01 | 17,74 |
| **09** | **Ariège** | 0,05 | 0,37 | 1,62 | 0,42 | 2,90 | 12,82 |
| **10** | **Aube** | 0,05 | 0,33 | 1,46 | 0,46 | 3,18 | 14,05 |
| **11** | **Aude** | 0,05 | 0,34 | 1,50 | 0,25 | 1,74 | 7,72 |
| **12** | **Aveyron** | 0,05 | 0,34 | 1,49 | 0,27 | 1,85 | 8,20 |
| **13** | **Bouches-du-Rhône** | 0,03 | 0,21 | 0,95 | 0,25 | 1,73 | 7,67 |
| **14** | **Calvados** | 0,05 | 0,38 | 1,67 | 0,50 | 3,46 | 15,32 |
| **15** | **Cantal** | 0,04 | 0,29 | 1,30 | 0,22 | 1,49 | 6,57 |
| **16** | **Charente** | 0,06 | 0,40 | 1,75 | 0,56 | 3,83 | 16,96 |
| **17** | **Charente-Maritime** | 0,06 | 0,44 | 1,94 | 0,49 | 3,40 | 15,03 |
| **18** | **Cher** | 0,06 | 0,40 | 1,78 | 0,33 | 2,25 | 9,97 |
| **19** | **Corrèze** | 0,06 | 0,42 | 1,88 | 0,43 | 2,98 | 13,19 |
| **2A** | **Corse-du-Sud** | 0,04 | 0,26 | 1,14 | 0,27 | 1,89 | 8,35 |
| **2B** | **Haute-Corse** | 0,03 | 0,20 | 0,87 | 0,20 | 1,39 | 6,14 |
| **21** | **Côte-d'Or** | 0,03 | 0,22 | 0,98 | 0,48 | 3,30 | 14,62 |
| **22** | **Côtes-d'Armor** | 0,08 | 0,57 | 2,54 | 0,69 | 4,73 | 20,92 |
| **23** | **Creuse** | 0,07 | 0,48 | 2,11 | 0,44 | 3,04 | 13,47 |
| **24** | **Dordogne** | 0,06 | 0,43 | 1,90 | 0,55 | 3,76 | 16,65 |
| **25** | **Doubs** | 0,05 | 0,34 | 1,50 | 0,38 | 2,62 | 11,59 |
| **26** | **Drôme** | 0,04 | 0,29 | 1,30 | 0,38 | 2,63 | 11,62 |
| **27** | **Eure** | 0,05 | 0,35 | 1,53 | 0,52 | 3,56 | 15,77 |
| **28** | **Eure-et-Loir** | 0,05 | 0,32 | 1,40 | 0,49 | 3,37 | 14,93 |
| **29** | **Finistère** | 0,07 | 0,47 | 2,08 | 0,59 | 4,04 | 17,86 |
| **30** | **Gard** | 0,04 | 0,29 | 1,28 | 0,30 | 2,06 | 9,11 |
| **31** | **Haute-Garonne** | 0,02 | 0,16 | 0,71 | 0,18 | 1,21 | 5,35 |
| **32** | **Gers** | 0,05 | 0,33 | 1,44 | 0,39 | 2,68 | 11,85 |
| **33** | **Gironde** | 0,04 | 0,27 | 1,19 | 0,45 | 3,11 | 13,76 |
| **34** | **Hérault** | 0,04 | 0,30 | 1,34 | 0,36 | 2,45 | 10,86 |
| **35** | **Ille-et-Vilaine** | 0,06 | 0,38 | 1,70 | 0,48 | 3,28 | 14,50 |
| **36** | **Indre** | 0,07 | 0,46 | 2,04 | 0,49 | 3,38 | 14,94 |
| **37** | **Indre-et-Loire** | 0,05 | 0,33 | 1,48 | 0,52 | 3,56 | 15,73 |
| **38** | **Isère** | 0,03 | 0,23 | 1,01 | 0,34 | 2,34 | 10,35 |
| **39** | **Jura** | 0,05 | 0,36 | 1,57 | 0,49 | 3,40 | 15,05 |
| **40** | **Landes** | 0,05 | 0,34 | 1,50 | 0,29 | 1,99 | 8,79 |
| **41** | **Loir-et-Cher** | 0,06 | 0,38 | 1,69 | 0,35 | 2,42 | 10,71 |
| **42** | **Loire** | 0,05 | 0,31 | 1,37 | 0,44 | 3,03 | 13,39 |
| **43** | **Haute-Loire** | 0,06 | 0,38 | 1,68 | 0,41 | 2,83 | 12,51 |
| **44** | **Loire-Atlantique** | 0,05 | 0,32 | 1,40 | 0,19 | 1,31 | 5,78 |
| **45** | **Loiret** | 0,05 | 0,31 | 1,39 | 0,47 | 3,26 | 14,45 |
| **46** | **Lot** | 0,05 | 0,34 | 1,49 | 0,42 | 2,89 | 12,80 |
| **47** | **Lot-et-Garonne** | 0,05 | 0,35 | 1,56 | 0,42 | 2,91 | 12,87 |
| **48** | **Lozère** | 0,04 | 0,28 | 1,24 | 0,30 | 2,10 | 9,28 |
| **49** | **Maine-et-Loire** | 0,05 | 0,35 | 1,53 | 0,40 | 2,78 | 12,29 |
| **50** | **Manche** | 0,08 | 0,53 | 2,35 | 0,62 | 4,24 | 18,77 |
| **51** | **Marne** | 0,05 | 0,33 | 1,45 | 0,53 | 3,65 | 16,14 |
| **52** | **Haute-Marne** | 0,06 | 0,40 | 1,77 | 0,57 | 3,90 | 17,26 |
| **53** | **Mayenne** | 0,06 | 0,43 | 1,89 | 0,47 | 3,26 | 14,44 |
| **54** | **Meurthe-et-Moselle** | 0,04 | 0,31 | 1,37 | 0,31 | 2,13 | 9,43 |
| **55** | **Meuse** | 0,06 | 0,43 | 1,89 | 0,52 | 3,59 | 15,88 |
| **56** | **Morbihan** | 0,07 | 0,51 | 2,28 | 0,52 | 3,59 | 15,90 |
| **57** | **Moselle** | 0,03 | 0,23 | 1,04 | 0,28 | 1,93 | 8,55 |
| **58** | **Nièvre** | 0,07 | 0,45 | 1,99 | 0,47 | 3,26 | 14,44 |
| **59** | **Nord** | 0,05 | 0,34 | 1,49 | 0,68 | 4,65 | 20,56 |
| **60** | **Oise** | 0,04 | 0,29 | 1,29 | 0,63 | 4,31 | 19,06 |
| **61** | **Orne** | 0,07 | 0,45 | 2,01 | 0,56 | 3,84 | 16,98 |
| **62** | **Pas-de-Calais** | 0,06 | 0,42 | 1,88 | 0,64 | 4,38 | 19,37 |
| **63** | **Puy-de-Dôme** | 0,05 | 0,34 | 1,50 | 0,22 | 1,51 | 6,67 |
| **64** | **Pyrénées-Atlantiques** | 0,05 | 0,31 | 1,38 | 0,18 | 1,25 | 5,53 |
| **65** | **Hautes-Pyrénées** | 0,05 | 0,32 | 1,42 | 0,42 | 2,90 | 12,83 |
| **66** | **Pyrénées-Orientales** | 0,05 | 0,36 | 1,57 | 0,29 | 2,01 | 8,91 |
| **67** | **Bas-Rhin** | 0,03 | 0,19 | 0,83 | 0,19 | 1,27 | 5,64 |
| **68** | **Haut-Rhin** | 0,04 | 0,27 | 1,18 | 0,19 | 1,28 | 5,65 |
| **69** | **Rhône** | 0,02 | 0,12 | 0,53 | 0,34 | 2,36 | 10,44 |
| **70** | **Haute-Saône** | 0,05 | 0,37 | 1,65 | 0,56 | 3,87 | 17,14 |
| **71** | **Saône-et-Loire** | 0,06 | 0,42 | 1,87 | 0,52 | 3,60 | 15,93 |
| **72** | **Sarthe** | 0,07 | 0,51 | 2,26 | 0,33 | 2,29 | 10,12 |
| **73** | **Savoie** | 0,04 | 0,26 | 1,14 | 0,30 | 2,10 | 9,27 |
| **74** | **Haute-Savoie** | 0,04 | 0,25 | 1,09 | 0,24 | 1,64 | 7,27 |
| **75** | **Paris** | 0,01 | 0,07 | 0,33 | 0,19 | 1,34 | 5,91 |
| **76** | **Seine-Maritime** | 0,05 | 0,32 | 1,41 | 0,67 | 4,60 | 20,35 |
| **77** | **Seine-et-Marne** | 0,03 | 0,20 | 0,87 | 0,35 | 2,38 | 10,51 |
| **78** | **Yvelines** | 0,03 | 0,21 | 0,91 | 0,28 | 1,92 | 8,50 |
| **79** | **Deux-Sèvres** | 0,06 | 0,40 | 1,76 | 0,44 | 3,03 | 13,42 |
| **80** | **Somme** | 0,06 | 0,39 | 1,75 | 0,96 | 6,63 | 29,32 |
| **81** | **Tarn** | 0,05 | 0,32 | 1,43 | 0,35 | 2,43 | 10,75 |
| **82** | **Tarn-et-Garonne** | 0,04 | 0,29 | 1,26 | 0,36 | 2,45 | 10,84 |
| **83** | **Var** | 0,05 | 0,35 | 1,56 | 0,41 | 2,81 | 12,43 |
| **84** | **Vaucluse** | 0,05 | 0,29 | 1,29 | 0,45 | 3,09 | 13,67 |
| **85** | **Vendée** | 0,05 | 0,36 | 1,58 | 0,39 | 2,68 | 11,87 |
| **86** | **Vienne** | 0,05 | 0,33 | 1,48 | 0,37 | 2,55 | 11,29 |
| **87** | **Haute-Vienne** | 0,05 | 0,37 | 1,64 | 0,40 | 2,73 | 12,08 |
| **88** | **Vosges** | 0,06 | 0,43 | 1,92 | 0,64 | 4,43 | 19,59 |
| **89** | **Yonne** | 0,06 | 0,38 | 1,67 | 0,41 | 2,79 | 12,35 |
| **90** | **Territoire de Belfort** | 0,04 | 0,29 | 1,27 | 0,59 | 4,03 | 17,81 |
| **91** | **Essonne** | 0,03 | 0,19 | 0,86 | 0,31 | 2,34 | 10,36 |
| **92** | **Hauts-de-Seine** | 0,02 | 0,15 | 0,67 | 0,24 | 1,68 | 7,44 |
| **93** | **Seine-Saint-Denis** | 0,01 | 0,09 | 0,41 | 0,18 | 1,23 | 5,43 |
| **94** | **Val-de-Marne** | 0,01 | 0,08 | 0,35 | 0,24 | 1,62 | 7,17 |
| **95** | **Val-d'Oise** | 0,03 | 0,18 | 0,79 | 0,24 | 1,64 | 7,27 |

**Table S1 –** *Rates of deaths by* *attempted suicides per day, per week and per month for each department in metropolitan France. Averages over the entire study period (2009-2018 for suicide attempts and 2009-2015 for suicides).*
